# Professional dental cleaning in dogs: clinical routines among Swedish veterinarians and veterinary nurses

**DOI:** 10.1186/s13028-020-00559-7

**Published:** 2020-11-11

**Authors:** Karolina Brunius Enlund, Michaela Karlsson, Carl Brunius, Ragnvi Hagman, Odd Viking Höglund, Pia Gustås, Jeanette Hanson, Ann Pettersson

**Affiliations:** 1 Department of Clinical Sciences, Swedish University of Agricultural Sciences, Uppsala, Sweden; 2 Anicura Albano Animal Hospital, Stockholm, Sweden; 3 Department of Biology and Biological Engineering, Food and Nutrition Science, Chalmers University of Technology, Gothenburg, Sweden

**Keywords:** Veterinary dentistry, Recommendation, Periodontal disease, Survey, Questionnaire

## Abstract

**Background:**

Dental disease is very common in dogs and veterinary professional dental cleaning and examination, together with daily dental home care, is the foundation for good dental health. To our knowledge, no previous study has investigated professional dental cleaning routines in small animal veterinary practice. A validated questionnaire survey was distributed to all veterinarians and veterinary nurses with registered e-mail addresses in the Swedish national registry (veterinarians; n = 3657, veterinary nurses; n = 1650). Response rates were 32% for veterinarians (V) and 38% for veterinary nurses (VN).

**Results:**

In total, 73% (V)/96% (VN) of respondents reported that professional dental cleaning was performed at their work place under general anesthesia, and 27% (V)/18% (VN) that dental cleaning was performed under sedation. Of the respondents, 43% (V)/96% (VN) considered regular dental cleaning under general anesthesia fairly or very important, and 49% (V)/47% (VN) stated that it was sometimes important for good dental health in dogs. A majority of respondents, 84% (V)/97% (VN), reported that dental extractions were performed at their clinic, and 72% (V)/90% (VN) had access to dental radiography equipment.

**Conclusion:**

A majority of Swedish veterinarians and veterinary nurses perform professional dental cleaning under general anesthesia with access to dental radiography equipment, in accordance with national and international recommendations. However, a considerable proportion of professional dental cleanings were performed under sedation only, and extractions performed without access to dental radiography equipment were common, suggesting several areas of improvement in the routines in Swedish veterinary clinics and hospitals. Our results clearly indicate the need for improved educational efforts to increase the awareness among veterinary health professionals regarding guidelines and official recommendations in canine dental care.

## Background

Periodontal disease is the most prevalent disease in dogs, with over 80% of dogs over 3 years of age affected [1–3]. Traumatic dento-alveolar injuries including tooth fractures are also very common in dogs, with one study reporting a prevalence of 26% [4]. Regular professional dental cleaning and evaluations at a veterinary clinic together with proper dental home care is the basis for good dental health in dogs.

There are multiple reasons for performing regular veterinary dental cleaning and thorough dental examinations. Early identification of periodontal disease is essential for prevention of disease progression. In addition, early evaluation of the oral cavity enables detection of disorders such as unerupted or malformed teeth and secondary damages to teeth and soft tissue caused by malocclusion. Moreover, routine dental checkups provide an excellent opportunity for enhanced communication with dog owners regarding proper dental homecare, including tooth brushing routines [5, 6]. A first visit to the veterinary clinic for dental cleaning and checkup is recommended from 1 year of age for small and medium sized dogs and from 2 years of age for larger dogs, and thereafter to be scheduled based on individual needs [5].

State-of-the-art professional dental cleaning includes a complete oral assessment, supra- and subgingival scaling (ultrasonic and/or hand-scaling), polishing, dental radiographs, and formation of a treatment plan [6]. The procedure should be performed under general anesthesia with the animal intubated (endotracheal tube), and connected to an anesthetic circle, enabling a thorough cleaning (subgingival as well as supragingival) and dental examination while minimizing the risk of debris and aerosol entering the airways. Full mouth radiographs are recommended as part of the examination to further reduce the risk of missing potential pathological conditions. In conjunction with tooth extractions, pre- as well as post-operative radiographs should be acquired in order to detect and avoid complications [6]. Veterinarians are uniquely qualified to examine, diagnose and address dental disease in dogs. However, in Sweden, veterinary nurses (registered veterinary technicians, RVT) are qualified to anesthetize and perform dental cleaning. Furthermore, certain dental procedures may be delegated to a veterinary nurse by a veterinary surgeon [7–9].

Both the World Small Animal Veterinary Association (WSAVA) and The American Animal Hospital Association (AAHA) have recently published evidence-based general guidelines and recommendations concerning performance of complete dental prophylaxis [5, 6]. These guidelines are endorsed nationally by the Swedish Veterinary Dental Association (Svenska Sällskapet för Djurtandvård, SSDT) [10] and have been incorporated in the veterinary and veterinary nurse curricula at the Swedish University of Agricultural Sciences (SLU), which is the sole provider of education towards these qualifications in Sweden.

It is however unknown to what extent Swedish veterinary health professionals adhere to these guidelines. To our knowledge, no previous study has investigated if sedation and/or general anesthesia are used when performing professional dental cleaning in dogs. Moreover, it is unknown whether veterinary clinics have access to dental radiography equipment, or if different background factors such as occupation (veterinarian or veterinary nurse) size of clinic and year of graduation may affect professional dental cleaning routines performed in veterinary small animal practices.

The aim of the present study was therefore to study these professional dental cleaning routines in small animal clinical practice in Sweden, as well as veterinarians’ and veterinary nurses’ attitudes regarding the importance of regular professional dental cleaning. This was achieved by the use of a validated questionnaire survey.

## Methods

A questionnaire survey to veterinarians and veterinary nurses concerning dental care in dogs, was constructed and validated according to survey methodology guidelines [34]. The study was approved by the Regional Ethical Review Board in Uppsala (Dnr 2017/035).

Questions within the survey regarding veterinary health professionals’ attitudes and information routines on canine dental home care have been reported previously [11]. In the present study, we present results regarding reported routines when performing dental cleaning in dogs in veterinary clinics. More specifically, these questions related to: whether dental cleaning was performed; by what occupational category (veterinarians and/or veterinary nurses) dental cleaning and dental extractions were performed; whether sedation or general anesthesia was used, and with which anesthetic agent (inhalation anesthesia, total intravenous anesthesia, dissociative anesthesia); whether there was access to dental radiography equipment; and finally how the importance of regular dental cleaning at the veterinary clinic was perceived by veterinarians and veterinary nurses.

The question about perceived importance of regular professional dental cleaning was visible to all respondents, whereas the rest of the questionnaire was only visible to respondents who answered that they met dogs in their professional role as a veterinarian (n_V_ = 932) or veterinary nurse (n_VN_ = 567). However, due to respondent dropout the number of responses may differ between questions. The question concerning type of anesthesia was only visible to respondents whose clinic performed dental cleaning under general anesthesia (n_V_ = 628; n_VN_ = 519). The questions concerning dental extractions and access to a dental x-ray unit is reported for respondents who answered that professional dental cleaning was performed at their workplace (n_V_ = 695; n_VN_ = 526). Size of clinic was measured as number of full-time employed veterinarians (1, 2, 3–5, 6–10, 11–30 or more than 30 veterinarians).

### Study design

The study design is described in detail elsewhere [34]. In brief, target groups consisted of all registered veterinarians (V; n = 4081), and all registered licensed veterinary nurses (VN; n = 1814) in Sweden. Sample frames were veterinarians and licensed veterinary nurses with e-mail addresses registered with the Swedish Board of Agriculture (24 February 2017) (V; n = 3657, VN; n = 1650). Veterinarians were also contacted by text message to their mobile telephone numbers obtained from the same register.

The questionnaire surveys were adapted for use on personal computers, tablets and smart phones, using the web platform Netigate (Netigate AB, Stockholm, Sweden). The questionnaires were distributed using individual web-links and reminders were sent to non-responders after 8 and 17 days. Data collection started on 31 March and was completed on 30 April 2017. Responses were collected anonymously, and the questionnaire could only be answered once per link. Response rates were 32% for veterinarians (n = 1161) and 38% for veterinary nurses (n = 642). Complete responses (< 2 missing values among six introductory questions that should have been answered by all respondents) were obtained from 1114 veterinarians and 609 veterinary nurses [11]. The length of the questionnaire for individual respondents depended on their answers and ranged from 17 to 49 questions. The questions were mainly closed, i.e. with fixed response options, and both nominal and ordinal data were collected [34].

### Statistics

Pretreatment of data was described in detail previously [34]. All statistical analysis was performed in the R open source statistical software v 3.5.1 [35]. The question whether dental cleaning was performed with ultrasonic scaler on dogs under sedation was analyzed for all respondents who reported meeting dogs in their profession (n = 1499) by logistic regression using the ‘glm’ function (family = binomial). The dichotomous variable whether the respondent performed the procedure themselves was used as the response, and with occupational category, gender, age category and clinic size as fixed factors. Results from the logistic regression are reported as odds ratios with 95% confidence intervals. Associations of anesthetics and dental radiography equipment with clinic size were investigated using chi-squared tests.

## Results

### Background characteristics

Background characteristics of respondents have been reported previously [11]. In brief, veterinarian respondents were 42.4 ± 12.8 years old (mean ± standard deviation) and veterinary nurse respondents 40.8 ± 9.6. There was a predominance of female veterinarians (77%) and veterinary nurses (97%). A majority of both veterinarians (62%) and veterinary nurses (51%) lived in an urban county (Stockholm, Skåne, Västra Götaland). In Sweden, 1984–1997 veterinary nursing was a 1-year university education, between 1998 and 2008 it was 2 years long and in 2009 it became a 3 year bachelor degree. Year of degree correlated strongly with age (r = 0.82) and 34% of veterinarians and 8% of veterinary nurses had received their degree prior to 2000. Many professionals, 62% of the veterinarians and 89% of the veterinary nurses, often encountered dogs in their professional role, and 73% of the veterinarians and 96% of the veterinary nurses worked in a pet clinic or animal hospital for dogs, cats and smaller animals. Of these, 26% of veterinarians and 36% of veterinary nurses worked at a clinic with 11 or more employed veterinarians [11].

### Survey results

#### Routines

The majority of veterinarians (73%) and veterinary nurses (96%) reported that dental cleaning was performed under general anesthesia at their workplace (Table 1), with 29% stating that only veterinarians, 30% that only veterinary nurses, and 41% that both veterinarians and veterinary nurses performed this procedure. Of these respondents, 33% of veterinarians and 59% of veterinary nurses reported performing dental cleaning under general anesthesia themselves.

**Table 1:** Routines and equipment in veterinary clinics regarding professional dental cleaning and dental radiography in dogs

		Veterinarians	Veterinary nurses
Is dental cleaning performed with ultrasonic scaler on dogs under general anesthesia at your workplace? (n_V_ = 865; n_VN_ = 542)	Yes	628 (73%)	519 (96%)
	No	183 (21%)	15 (3%)
	Don’t know/choose not to answer	54 (6%)	8 (1%)
What type of general anesthesia is used when performing dental cleaning with ultrasonic scaler? *(Question visible only if dental cleaning was performed under general anesthesia)* (several answers could be given) (n_V_ = 628; n_VN_ = 519)	Inhalation anesthesia	425 (68%)	417 (80%)
	Total intravenous anesthesia	196 (31%)	123 (24%)
	Dissociative anesthesia	86 (14%)	51 (10%)
	Don’t know/other	16 (3%)	14 (3%)
Is dental cleaning performed with ultrasonic scaler on dogs under sedation (e.g. dexmedetomidine/butorphanol) at your workplace? (n_V_ = 867; n_VN_ = 542)	Yes	230 (27%)	96 (18%)
	No	576 (66%)	425 (78%)
	Don’t know/choose not to answer	61 (7%)	21 (4%)
At your workplace: Are teeth sometimes extracted when performing a dental cleaning? *(Only reported for respondents who answered that professional dental cleaning was performed at the workplace)* (n_V_ = 695 ; n_VN_ = 526)	Yes	680 (98%)	519 (99%)
	No	12 (2%)	2 (0%)
	Don’t know	3 (0)	5 (0%)
Do you have access to a dental x-ray unit? *(Only reported for respondents who answered that professional dental cleaning was performed at the workplace)* (n_V_ = 695; n_VN_ = 526)	Yes	497 (72%)	471 (90%)
	No	193 (28%)	51 (10%)
	Don’t know	5 (1%)	4 (1%)

**Fig. 1:**
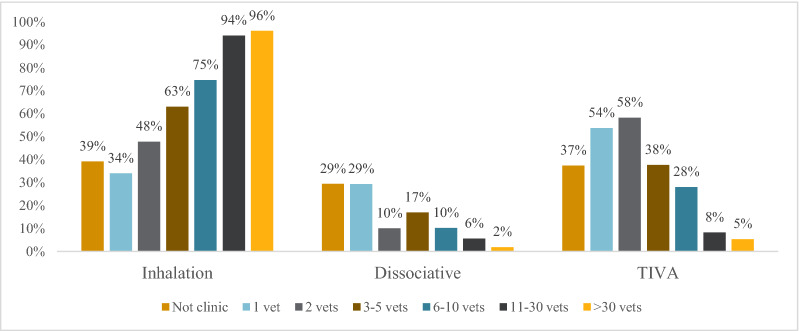
Type of general anesthesia used when performing dental cleaning with ultrasonic scaler. Reported as proportions of veterinarians and veterinary nurses per clinic size. Not clinic denotes V and VN who reported that they do not work in a small animal clinic/hospital. Several options could be specified, wherefore responses sum up to > 100%. This question was visible only if dental cleaning was reported to be performed under general anesthesia (n_V_ = 628; n_VN_ = 519)

The most common type of general anesthesia was inhalation anesthesia, followed by total intravenous anesthesia and dissociative anesthesia (Table 1). The use of total intravenous anesthesia (TIVA, e.g. propofol; P = 2.6 × 10^–26^) and dissociative anesthesia (ketamine; P = 3.3 × 10^–7^) associated inversely with clinic size (Fig. 1). Multiple responses were possible and 15% of respondents answered that they used more than one type of anesthesia.

**Fig. 2:**
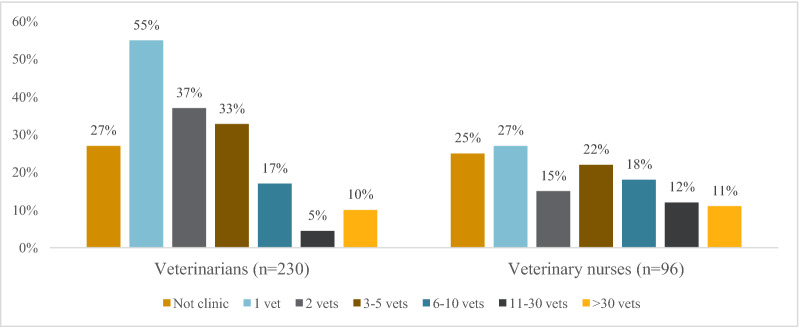
Professional dental cleaning performed with ultrasonic scaler under sedation (e.g. dexmedetomidine/butorphanol). Reported as proportions of veterinarians and veterinary nurses per clinic size. Not clinic denotes V and VN who reported that they do not work in a small animal clinic/hospital

In total 27% of veterinarians and 18% of veterinary nurses who regularly met dogs in their profession, stated that dental cleaning was performed under sedation at their workplace (Table 1 and Fig. 2). Of these, 51% stated that only veterinarians, 23% that only veterinary nurses, and 26% that both veterinarians and veterinary nurses performed the dental cleaning. Of these respondents, 16% of veterinarians and 11% of veterinary nurses reported performing dental cleaning under sedation themselves.

As many as 23% of veterinarians and 17% of the veterinary nurses responded that dental cleaning was performed both under sedation and general anesthesia at their workplace, and 24% of veterinarians and 2% of veterinary nurses stated that they did not perform dental cleaning under sedation or general anesthesia, at the work place.

**Fig. 3:**
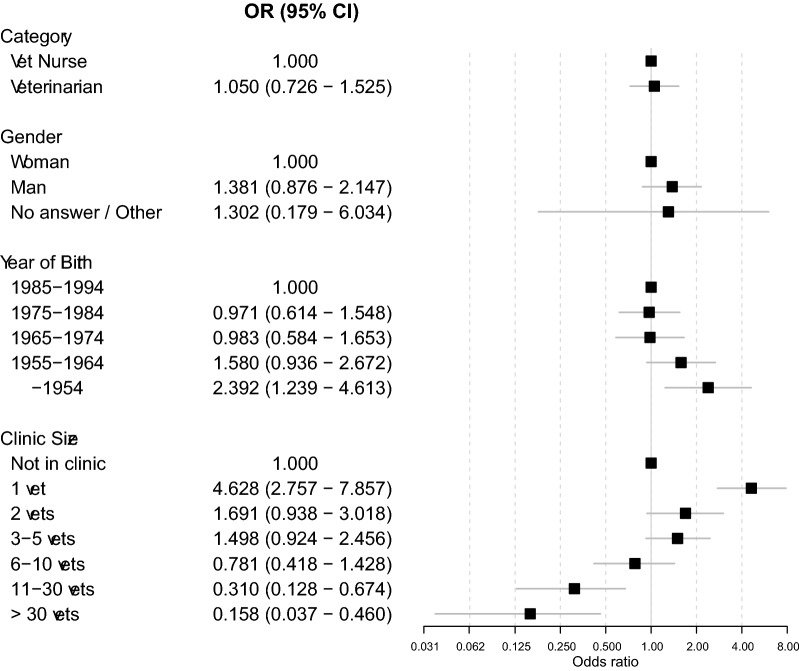
Associations of age group, gender, clinic size and occupational category with veterinary health professionals’ likelihood to perform dental cleaning under sedation (e.g. dexmedetomidine/butorphanol). Reported as odds ratio (OR) with 95% CI for respondents performing dental cleaning themselves (n_V_ = 143; n_VN_ = 61)

Dental cleaning performed under sedation was more common in smaller veterinary clinics and among older veterinary health professionals (Fig. 3).

Of veterinary health professionals reporting that professional dental cleaning was performed at their workplace, 98% of veterinarians and 99% of veterinary nurses reported that teeth were sometimes extracted during the procedure (Table 1). Of these respondents, 70% stated that only veterinarians, 4% that only veterinary nurses, and 26% that both veterinarians and veterinary nurses performed dental extractions.

#### Equipment

Of veterinary health professionals reporting that professional dental cleaning was performed at their workplace, 72% of veterinarians and 90% of veterinary nurses reported that they had access to dental radiography equipment. Access to such equipment was associated with increased clinic size (P < 2.2 × 10^–16^).

**Fig. 4:**
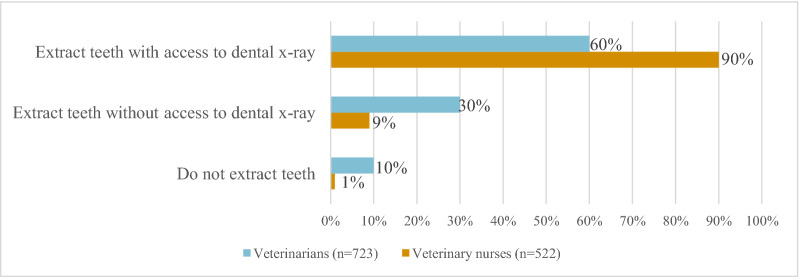
Performance of dental extractions in veterinary clinic with or without access to dental radiography equipment. Note that the profession refers to the respondent and not the occupational category performing extractions

Of respondents who reported dental extractions being performed at the veterinary clinic, 30% of veterinarians and 9% of veterinary nurses stated that they did not have access to dental radiography equipment at the clinic (Fig. 4).

#### Attitudes

**Fig. 5:**
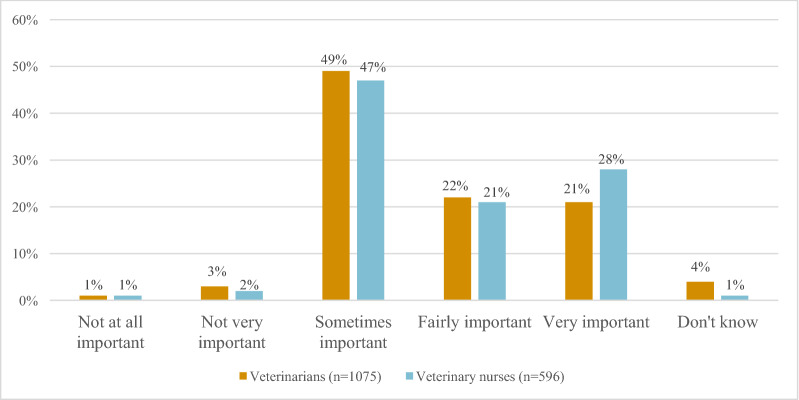
Perception of the importance of regular dental cleaning under anesthesia for good dental health in dogs

Regular dental cleaning under general anesthesia was considered important (fairly or very) for good dental health in dogs by 43% of veterinarians and 49% of veterinary nurses. Moreover, 49% of veterinarians and 47% of veterinary nurses, stated that it was sometimes important (Fig. 5).

## Discussion

Regular professional dental cleaning and examinations are important parts of pet animals’ preventive healthcare and has the potential to improve welfare in dogs. In comparison, prophylactic dental examinations are implemented for children from the age of three in Sweden [12]. The human dentistry profession in Sweden focuses on the establishment of proper dental homecare routines in order to prevent caries and the development of periodontal disease. Oral health is monitored throughout adolescence by state subsidized regular dental examinations and professional dental cleaning. Compliance in humans with the recommendations for tooth brushing, i.e. twice daily, is high in Sweden [13]. This is in stark contrast to dogs, where only 4% of dog owners brush their dog’s teeth daily [11]. Routine dental examinations and cleaning in dogs have the potential to achieve improved compliance also for daily tooth brushing in dogs. The AAHA recommendations for initiating routine dental check-ups in dogs from 1 to 2 years of age is in line with routines within human dentistry with the ultimate goal to prevent oral disease before irreversible damage has occurred. We have previously shown that dog owners attribute high importance to dogs’ dental health [14]. In relation to this, it is encouraging that up to 50% of veterinary health professionals in the present study considered it fairly or very important that regular professional dental cleaning be carried out at a veterinary clinic (Fig. 5). However, our results also indicate that many veterinary health professionals may not be aware of the importance of detecting oral anomalies and conveying recommendations of prophylactic routines regarding canine dental care early in the dog’s life.

### Professional dental cleaning under sedation versus general anesthesia

Guidelines for dental treatments state the importance of performing dental procedures under general anesthesia, with the dog intubated and connected to a closed circuit, to avoid the risk of aspiration of bacterial aerosol and debris as well as gastric contents [5, 6, 15, 16]. Intubation is generally not possible in solely sedated animals and the airways are therefore unprotected [6, 16]. Aspiration is a known risk in all anesthesia and sedation, as many sedative and anesthetic agents predispose for vomiting and gastroesophageal reflux [17, 18]. Aspiration can lead to pneumonia but can also give milder symptoms, so called silent aspiration, which may not need treatment [17, 19–22]. Results from one retrospective study suggests that aspiration complications seem to be rare [18]. The incidence of canine post-anesthetic aspiration pneumonia was only 0.17%, and dental procedures were not associated with an increased risk.

In addition to the potential risk of aspiration, proper supra- and subgingival cleaning of plaque and calculus as well as a thorough dental examination with probing of pockets and dental radiography is difficult to achieve in sedated dogs [6, 15]. Further, monitoring of cardiopulmonary functions may be difficult to uphold during procedures using solely sedation [6]. It was therefore encouraging that the majority of veterinary health professionals reported performing dental cleaning under general anesthesia, especially at larger clinics.

However, in contrast to AAHA and WSAVA recommendations, our results showed that dental cleaning on sedated animals was common, especially in smaller clinics. In the free text, respondents commented that they considered this to be satisfactory because they had not experienced any previous problems in conjunction with dental cleaning in sedated dogs (data not shown). Even though the guidelines are clear about the recommendation for general anesthesia, this suggests a need for more detailed investigations into the risk for aspiration during dental procedures in dogs, especially under sedation. Nevertheless, the results from our study suggest that educational efforts are needed among veterinary health professionals regarding general guidelines.

So-called anesthesia free dental cleaning, although the subject of much layman debate, is not considered an acceptable treatment for oral disease, partly for the same reason: It is impossible to perform a thorough dental examination in the fully conscious animal [5, 6, 23, 24]. Such procedures may instead lead to a false sense of security if owners believe that the removal of supragingival calculus in fact constitutes a medical benefit and additionally impose an increased risk of injuries to both dogs and professionals [5, 24].

Older veterinarians and veterinary nurses were more prone to perform dental cleaning under sedation even with adjustment for other factors, such as clinic size, in the statistical model. This may be related to veterinary clinicians not updating established routines regarding the handling of dental patients. It may also reflect progress in the veterinary dentistry education.

It was more common among veterinarians than veterinary nurses to state that professional dental cleaning was performed under sedation. A possible reason may be that a larger proportion of veterinarians work alone or in small clinics. The correlation between small clinics and dental cleaning performed under sedation only may also indicate a lack of equipment for inhalation anesthesia. In addition, smaller clinics may attract more cost-conscious animal owners compared to larger animal clinics or hospitals, which may further contribute to the use of the less expensive sedation procedure.

One possible explanation why veterinary health professionals use both sedation and general anesthesia in the same clinic for dental patients may be that dental cleaning under sedation is performed in dogs which are evaluated as having only minor dental problems, e.g. only dental calculus without signs of periodontal disease. Whereas in dogs that have previously shown signs of periodontal disease, have concurrent disease or are assessed as having more severe dental problems are treated under general anesthesia.

### Dental extractions and dental radiography

Dental extractions without access to dental radiography equipment were reported to be commonly performed by both veterinarians and veterinary nurses (Fig. 4). Unfortunately, complications to extractions are known to be common [25] and dental radiographs should precede all extractions, even seemingly simpler ones, to detect any abnormal structural deviations, such as root fractures, abnormal root formation, supernumerary roots or other pathologies that could affect the procedure [6]. Studies have shown that such abnormal radiographic findings are common [26, 27]. Apart from ensuring that no retained roots are present, postoperative radiographs may be used to confirm a successful extraction without other complications such as iatrogenic fractures. Our results suggest that this recommendation may not be known among all veterinarians and veterinary nurses and illustrates the need for improvement regarding dental extractions routines.

According to the present study, it is common practice that both veterinarians and veterinary nurses extract teeth. According to Swedish national guidelines dental extractions are considered surgical procedures, and should therefore be performed by a veterinarian according to legislation [7, 9]. However, extractions of mobile, single-rooted teeth may, after radiographic evaluation, be regarded as a simple procedure and may be delegated to a veterinary nurse [7]. In the USA, legislation varies from state to state and extractions by veterinary technicians may be allowed under supervision of a veterinary surgeon [28]. The American Veterinary Dental College (AVDC) states that “only veterinarians shall determine which teeth are to be extracted and perform extraction procedures” [29]. The Royal College of Veterinary Surgeons (RCVS) precludes veterinary nurses from performing dental extraction [28]. We observed that both veterinarians and veterinary nurses perform dental extractions, which may indicate that more complicated extractions were performed by the veterinarian whereas the veterinary nurse performed simple extractions. However, the data does not differentiate which type of extractions were executed by the different professions.

### The importance of regular professional dental cleaning

About half of both veterinarians and veterinary nurses stated that regular professional dental cleaning is only sometimes important (Fig. 5). We hypothesize that veterinary health professionals may not consider this to be important until dental problems have been confirmed. If this were the case, it would be unfortunate, since prevention of dental problems is always preferable to treatment of already existing disease [30]. There may also have been a tendency in the past to trivialize dental disease, thereby failing to acknowledge that un-noticed dental disease frequently leads to undue suffering and thus has a major impact on dogs’ well-being. Another possibility is that veterinary health professionals may deem professional dental cleaning to be more important in certain, especially smaller breeds which are known to be predisposed to periodontal disease [1, 14, 31–33]. However, all dogs are at risk of being affected by dental disease and regular professional dental cleaning and assessment should be a part of every dog’s life-long health plan [5].

The results from the present study provides a basis for future follow-up studies regarding veterinary health professionals’ attitudes and routines regarding dental treatment in dogs.

### Strengths and limitations

The study has several strengths: the sample size was large and collected responses are therefore likely to accurately reflect opinions and attitudes of the study populations. In addition, the representativity of the respondents to the target population was thoroughly investigated and found to be overall satisfactory, and state-of-the-art methods in survey construction and validation were applied to ensure high data quality [34].

The study also has limitations: The study was performed in a Swedish social and cultural context and results may therefore not be generalizable in an international context. Despite a meticulous validation procedure, questionnaire surveys are inevitably susceptible to bias, e.g. the risk that respondents may be more interested in the subject than the average.

The target population for the survey was individual veterinarians and veterinary nurses, and therefore respondents may in fact work in the same veterinary clinic/hospital (applies in particular to respondents who work in larger working units), wherefore the obtained results may not accurately reflect differences between clinics.

Further, the questionnaire did not include questions whether dogs were intubated or whether they were connected to a closed circuit, since these questions were deemed to be sensitive and may cause irritation and social desirability bias which may lead to unreliable results. Moreover, the questionnaire was not constructed exclusively to investigate sedation/anesthesia, dental radiography equipment and extraction routines, and therefore did not provide details on professional dental scaling procedures, such as whether hand scaling was performed, whether sub- as well as supragingival scaling was performed, if teeth were polished, and what type of dental extractions were performed by what professional category. Further studies are warranted to address these issues, as well as longitudinal studies to study dental care routines over time.

## Conclusions

A majority of Swedish veterinarians and veterinary nurses report that professional dental cleaning is performed under general anesthesia with access to dental radiography equipment, in accordance with national and international recommendations. However, the considerable proportion of professional dental cleanings performed under sedation only and extractions performed without access to dental radiography equipment suggests several areas in need of improvement in the routines of Swedish veterinary clinics and hospitals. Our results propose the need for educational efforts to increase the awareness among veterinary health professionals regarding guidelines and official recommendations in canine dental care.

## Data Availability

The data is available from the authors upon reasonable request. The data are not publicly available due to them containing information that could compromise research participant privacy.
